# Health Vulnerability Index for Disaster Risk Reduction: Application in Belt and Road Initiative (BRI) Region

**DOI:** 10.3390/ijerph16030380

**Published:** 2019-01-29

**Authors:** Emily Yang Ying Chan, Zhe Huang, Holly Ching Yu Lam, Carol Ka Po Wong, Qiang Zou

**Affiliations:** 1 Collaborating Centre for Oxford University and CUHK for Disaster and Medical Humanitarian Response (CCOUC), JC (Jockey Club) school of Public Health and Primary Care, Faculty of Medicine, Chinese University of Hong Kong, Hong Kong, China; huangzhe@cuhk.edu.hk (Z.H.); hollylam@cuhk.edu.hk (H.C.Y.L.); wongcarol@cuhk.edu.hk (C.K.P.W.); 2 Nuffield Department of Medicine, University of Oxford, Oxford OX3 7BN, UK; 3 François-Xavier Bagnoud Center for Health & Human Rights, Harvard University, Boston, MA 02138, USA; 4 Institute of Mountain Hazards and Environment, Chinese Academy of Science, Chengdu 610041, China; zouqiang@imde.ac.cn

**Keywords:** Health vulnerability, Health-EDRM, disaster risk, Silk Road Economic Belt, map, Belt and Road Initiative

## Abstract

Despite the importance of health vulnerability in disaster risk assessment, most of the existing disaster vulnerability indicators only emphasize economic and social vulnerability. Important underlying health risks such as non-communicable disease are not included in vulnerability measures. A three-phase methodology approach was used to construct a disaster risk model that includes a number of key health indicators which might be missing in global disaster risk analysis. This study describes the development of an integrated health vulnerability index and explains how the proposed vulnerability index may be incorporated into an all-hazard based disaster risk index in the Belt and Road Initiative (BRI), also known as the “Silk Road Economic Belt”, region. Relevant indicators were identified and reviewed in the published literature in PubMed/Medline. A two-stage dimension reduction statistical method was used to determine the weightings of relevant dimensions to the construction of the overall vulnerability index. The proposed final health vulnerability index included nine indicators, including the proportion of the population below 15 and above 65 years, under-five mortality ratio, maternal mortality ratio, tuberculosis prevalence, age-standardized raised blood pressure, physician ratio, hospital bed ratio, and coverage of the measles-containing-vaccine first-dose (MCV1) and diphtheria tetanus toxoid and pertussis (DTP3) vaccines. This proposed index, which has a better reflection of the health vulnerability in communities, may serve as a policy and implementation tool to facilitate the capacity-building of Health-Emergency Disaster Risk management (Health-EDRM).

## 1. Introduction

Disasters have brought huge losses in human health and the economy globally. According to Economic Losses, Poverty & Disasters, 1998–2017 issued by the Centre for Research on the Epidemiology of Disasters and United Nations Office for Disaster Risk Reduction in 2018, climate-related and geophysical disasters alone have taken lives from 1.3 million people, and have affected 4.4 billion people in the world between 1998–2017. The report also highlighted a global direct economic loss of USD 2908 billion due to disasters within the same period [1]. Asia, similar to previous years, suffered from the highest disaster occurrence (more than 40% of the total) [2], while China, India, Indonesia, and the Philippines were four of the top five countries that were most frequently hit by natural disasters over the last decade [3]. Due to climate change, both the frequency and intensity of disasters have been predicated to increase in the 21st century [4]. Relevant risk assessment tools and disaster risk reduction plans are important for saving lives and reducing losses in the future.

Understanding disaster risk in all its dimensions is the first priority for Disaster Risk Reduction action in the Sendai Framework, which was the first major agreement endorsed by the United Nations (UN) General Assembly on Disaster Risk Reduction for policies and practices for disaster risk management [5]. Disaster risk can be conceptualized as a function of hazard, exposure, and vulnerability [6]. According to UNISDR [7], risk is defined as the harmful consequences resulting from interactions between hazards, exposure, and vulnerable conditions. Hazard refers to dangerous phenomena that may cause negative health impacts; exposure refers to the people who are present in hazard zones and subject to potential health losses; vulnerability refers to the characteristics and circumstances of a community that make it susceptible to the damaging effects of a hazard. Disaster risk assessment can be understood as quantifying these three components among the population.

There are major technical gaps in how to describe vulnerability, particularly to health risks, when constructing disaster risk indexes [8]. Existing vulnerability indicators/indexes mostly focus on economic and social vulnerability [9,10,11]. Most health vulnerability indexes were developed after 2010, and were related to human health vulnerability toward climate-related disasters such as heat wave [12,13,14], flooding [15], dengue fever [16], and climate change [17]. In addition, as the data used for index construction were largely based on the country’s own capacity in data collection, multi-country comparisons are often difficult, as countries may have different data collection methods and capacities. The Index For Risk Management (INFORM), a collaborative work with the United Nations, and the World Risk Index, a joint work with the Integrated Research on Disaster Risk (IRDR), are sophisticated global disaster risk indexes that have accounted for health vulnerability [18,19]. However, the current indexes do not include important health-affecting factors such as chronic diseases. Chronic disease is an important aspect to be considered in disaster risk management, as discontinuous treatment and medicine, which is possible during a disaster event, can lead to adverse health consequences among chronic disease patients. For instance, the provision of insulin may sustain the well-being and survival of diabetes patients [20].

Under the influence of globalization, the spread of health risks is borderless, and the prevention and control of health emergencies (e.g., disasters) need to be managed collaboratively. China’s Belt and Road Initiative (BRI), also known as the “Silk Road Economic Belt”, was initiated in 2013 and aimed to connect the Asian, European, and African continents and their adjacent seas, and establish and strengthen partnerships among the countries along the Belt and Road [21]. Among these BRI countries, various types of disasters occur frequently, and the widespread damage and destruction caused by disasters seriously disrupts the functioning of a society, and poses a major socio-economic development challenge for the Belt and Road Initiative region. The BRI also provides a health cooperation platform to handle regional health emergencies, offers medical assistance, and disseminates experience in the field of health care [22]. Understanding disaster risk and vulnerability for the countries along the Belt and Road is crucial for resource allocation. Yet, current available health vulnerability indexes may not apply to the countries within Belt and Road Initiative.

Health-Emergency Disaster Risk Management (Health-EDRM) is an academic paradigm representing the intersection of health and disaster risk reduction that covers the systematic analysis and management of health risks surrounding emergencies and disasters [23]. This study falls into the primary Health-EDRM intervention category (prevention/preparedness) in the system (country) level.

The objective of this paper is to describe the development of a health vulnerability index that aims to be incorporated into the vulnerability index, and might be applied to the use of the all-hazard based disaster risk index in the BRI region. The developed index described in this study used open access data and proposed indicators that are available in most countries and make disaster risk comparison between countries possible. The proposed method can also be adopted by regions or populations that were not included in this study. The findings from this study provide evidence to support disaster risk reduction in the BRI regions, and serve as a basis for the development of a population-based disaster risk assessment tool.

## 2. Materials and Methods

A three-phase methodology approach was used to develop the final disaster risk model. Phase 1 of the approach focuses on the development of the health vulnerability index, which includes an extensive literature review to identify the relevant published indicators to construct health vulnerability. Phase 2 involves a two-stage dimension reduction statistical method to identify the weighting for the indicators that were included for the health vulnerability index development. Phase 3 aims to create the final disaster risk index by combining the three main component indexes (health vulnerability index, exposure, and hazard index), which can be described in the following equation: Risk = Exposure × Hazard × Vulnerability [6]. The health vulnerability index is combined with existing exposure and hazard indexes to form a disaster risk index at the national level. The exposure and hazard indexes were based and accessed through the Institute of Mountain Hazards and Environment at the Chinese Academy of Science (http://english.imde.cas.cn). As the mechanisms and the development of the two indexes were out of the scope of this study, they were not included in this paper’s discussion.

### 2.1. Phase I

#### Data Scoping and Variable Selection

Variable selection criteria include: (1) any indicator that is conceptually relevant to health vulnerability or may capture the Health-EDRM risks of the community; (2) indicators that have been identified/suggested in relevant literatures or organizations (e.g., the World Health Organization (WHO), UN, INFORM model [18] and World Risk Index [19]), and (3) indicators that are available for open access from reliable sources (e.g., WHO and the World Bank) for all of the study regions. Since subsequent factor analysis cannot be performed with missing values, countries with missing values were excluded in the subsequent analysis. The countries/areas along the BRI region and countries/areas included in the analysis were listed in the supplementary material A1.

### 2.2. Phase II

#### Statistical Model for the Health Vulnerability Index

As this study made no assumption on the weighting for indicators, in order to determine the weightings and explore the importance of the underlying dimensions to the overall vulnerability, a two-stage dimension reduction statistical method was used. This method also increases robustness [10] and allows monitoring changes in the weighting of indicators over time. In stage one, factor analysis (FA) was used as the primary statistical procedure for dimension reduction. The observed and correlated indicators were assumed to be adequately explained by a lower number of unobserved and uncorrelated factors. Stage two modeling was based on the result of FA; the selected health indicators were used to produce a more compact representation of the indicators (factors).

Stage 1: Selected indicators are normalized and included in the FA analysis. The matrix of factor loadings was estimated via the maximum likelihood method, and the number of factors that is extracted should contribute cumulatively to the explanation of the overall variance by more than 60% [24]. The Chi-square test was used to examine whether the number of factors, k, are sufficient to account for the observed covariance [25]. A non-significant Chi-square test result (*p* ≥ 0.05) indicates that k is sufficient to explain the observed covariance. The explanation power increases when k increases. To obtain the most efficient model, the smallest k that yielded a non-significant Chi-square test was chosen. The initial k tried was one, and then, k was increased by one at a time. Then, the process is repeated until the p-value of the Chi-square test ≥ 0.05 [25]. Factors identified from FA are sometimes expressed as a compound with a relative large number of non-zero weighting indicators, which may make factor interpretation hard. To make interpretations of factors easier, Varimax rotation [26] was conducted to obtain as few large loadings and as many near-zero loadings as possible.

Stage 2: The development of the Health Vulnerability Index (HVI) is based on the latent factors derived from the FA. Each latent factor has factor loading on every health indicator, measuring the correlation between the health factor and the health indicator. The construction of the weights of the selected health indicators is from the rotated matrix of factor loadings [24,27]: (1) the proportion on each latent factor of the total unit variance was extracted; (2) the intermediate weights of all of the health indicators were calculated from the factor loadings corresponding to the latent factor; (3) the proportion on each latent factor is multiplied by the intermediate weights of all the health indicators on each latent factor to generate the weights for all the selected health indicators. Finally, the weights were multiplied by the corresponding standardized health indicator, and were added together for every country’s HVI. A higher value indicates a more vulnerable country. The HVI value of each country involved was categorized into five HVI clusters using the equal interval method for data presentation in the form of a vulnerability map. A five-level scale was selected, as it provides a good balance between risk-level differentiation and clarity, and has been widely adopted in risk-level presentations [28,29]. R version 3.4.1 and ArcMap version 10.4.1 were used.

### 2.3. Phase III

#### Disaster Risk Index Model

##### Exposure and Hazard

The exposure and hazard index were provided by the working dataset of The Institute of Mountain Hazards and Environment at The Chinese Academy of Science. The exposure index was estimated by using the population density data from the Socioeconomic Data and Applications Center [30] as a proxy. For hazard, the frequency of natural disaster was applied. Both indexes were in a pixel-based format, and can be illustrated using a map.

##### Disaster Risk Index

Raster values from the Exposure index, the Hazard index, and the Vulnerability index were multiplied to generate the final Risk index. Since both the exposure and the hazard indexes are pixel-based data, the vulnerability index was transformed from the country-based format to the pixel-based using ArcMap before combining with the other two indexes to form the final risk index.

Calculation of Risk (Risk = Exposure × Hazard × Vulnerability)(1)

Logarithm transformations were applied for skewed data, including the exposure index and the hazard index. The log-transformed indexes and the vulnerability index were then transformed to a 0–1 scale using min–max normalization. The final disaster risk index was calculated by multiplying the three transformed components with equal weight (Formula (1)) [18,19]. The results were presented in the form of map in a scale with five risk levels.

## 3. Results

### 3.1. Key Indicators of Vulnerability

Based on the three evaluation criteria, nine health indicators were identified and included in the final index development. Table 1 describes the key health indicators included.

**Figure 1:**
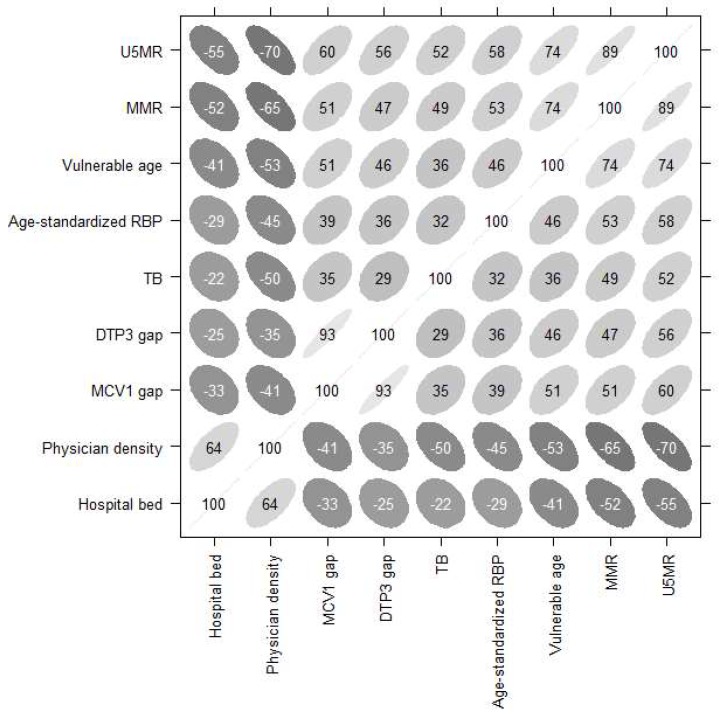
Correlation matrix of the proposed nine health indicators. Note: The figure depicts each correlation by an ellipse whose shape tends toward a line with a slope of one (or −1) for correlation coefficients near one (or −1), and toward a circle for a correlation coefficient near zero. In addition, 100 times the correlation coefficient is printed inside the ellipse (significance level at α = 0.05).

The Health Vulnerability Index was constructed for the 147 countries along the Belt and Road region. Indicators one, six, and seven are related to population structure and health status; indicators four and five are used to monitor immunization services, which are good indictors of health system performance; indicators two and three are leading indicators of the level of child and maternal health, as well as the overall development in countries; and indictors eight and nine measure the availability of healthcare, and are important indicators of disaster coping capacity. The correlations between the nine health indicators were shown in Figure 1. All of the correlations presented are statistically significant (all *p*-values < 0.01).

### 3.2. Underlying Dimensions of Health Vulnerability

**Figure 2:**
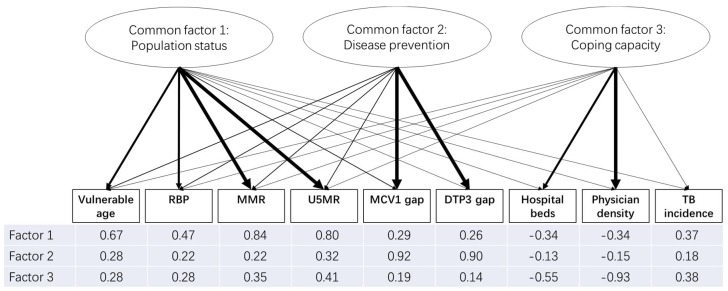
Factor loadings of the three latent factors. Note: Factor loadings are printed under the corresponding indicator. They are also indicated by the thickness of the arrow linking the factor and the indicator: the thicker the arrow, the higher the factor loading. Arrows are not shown if the absolute value of the factor loading is less than 0.2. Vulnerable age: people aged 0–14 or/and 65+ (%); RBP: Age-standardized raised blood pressure prevalence (%); MMR: Maternal mortality ratio (per 100,000 live births); U5MR: Under-five mortality rate (per 1000 live births); MCV1 gap: MCV1 Coverage Gap (%); DTP3 gap: DTP3 Coverage Gap (%); Hospital beds: Hospital beds density (per 10,000 population); Physician density: Physicians density (per 1000 population); TB incidence: Incidence of tuberculosis (per 100,000 population).

The results of the Chi-square test for the sufficiency of the number of factors suggested that a three-factor solution was adequate to account for the observed covariances in the data among the 147 countries. Both of the eigenvalues of the three-factor solution were larger than one, and the three factors counted for about 71% of the total variance. Factor loadings after rotation are shown in Figure 2.

Since factor one is dominated by the maternal mortality ratio (per 100,000 live births) (MMR, 0.84) and the under-five mortality rate (per 1000 live births) (U5MR, 0.80), and moderately affected by vulnerable age (0.67) and age-standardized raised blood pressure prevalence (RBP) (0.47), this factor was labeled the “population status” factor. The second factor has its highest loadings on the measles-containing-vaccine first-dose (MCV1) gap (0.92) and diphtheria tetanus toxoid and pertussis (DTP3) gap (0.90), which was labeled the “disease prevention” factor. The third factor was highly correlated with physician density (0.92) and moderately with hospital beds (0.90), so it was labeled the “coping capacity” factor.

### 3.3. Factor Scores of Countries

**Figure 3:**
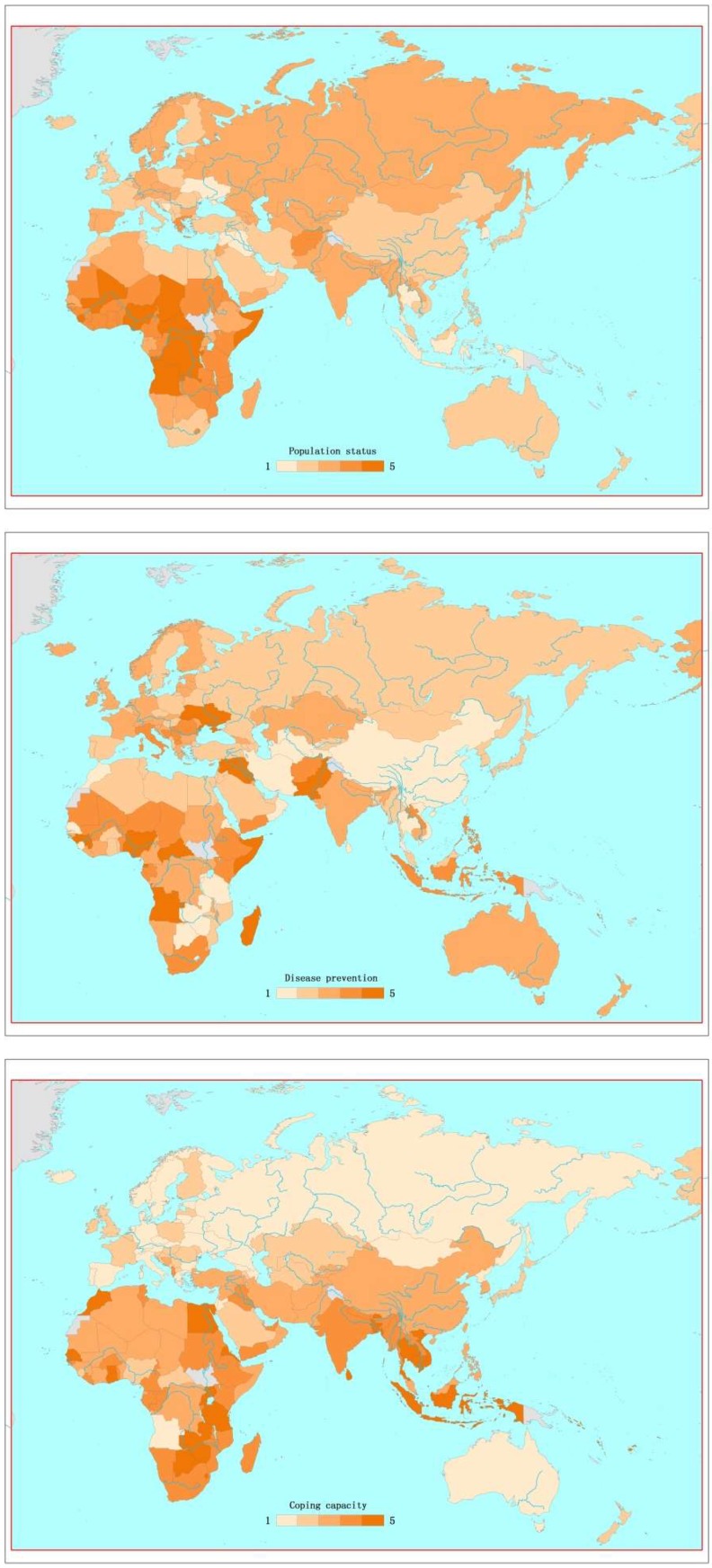
Factor scores of countries along the Belt and Road Initiative (BRI) region. Note: deeper color indicates a higher factor score and greater vulnerability.

The estimated scores of factors one to three for each country were calculated and categorized into five levels, as shown in Figure 3. Considering factor one, which reflects population status, Sierra Leone, Chad, and the Central African Republic are the most vulnerable countries, whilst Ukraine was shown to be the least vulnerable among all of the studied countries. For the second factor, Equatorial Guinea and Ukraine are prominent, because they had low MCV1 and DTP3 immunization coverage. For factor three, Thailand, the Solomon Islands, and Indonesia were at the highest end of the scale.

### 3.4. Health Vulnerability Index of Countries

**Figure 4:**
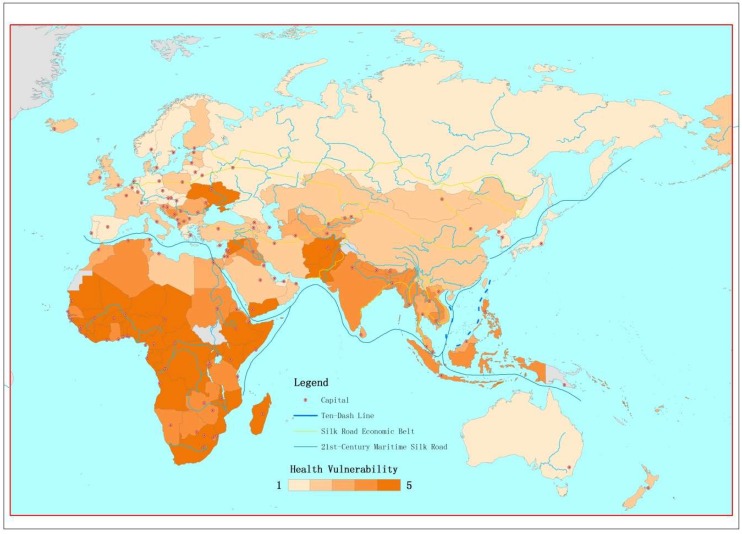
Health Vulnerability Index of countries along the Belt and Road Initiative (BRI) region. Note: deeper color indicates greater vulnerability.

The development of the HVI was based on the FA model above, which captured the relative weights of the six health indicators. Weights for vulnerable age, RBP, MMR, U5MR, MCV1 gap, DTP3 gap, hospital beds, physician density, and incidence of tuberculosis (TB, per 100,000 population) were 0.10, 0.05, 0.14, 0.14, 0.15, 0.14, 0.07, 0.16, and 0.05, respectively. Greece, the Republic of Korea, and Belarus were the three least vulnerable countries, whereas countries labeled in the darkest color (the most vulnerable) were clustered in Africa, such as Somalia, the Central African Republic, and Chad (Figure 4).

### 3.5. Disaster Risk Mapping in Silk Road

**Figure 5:**
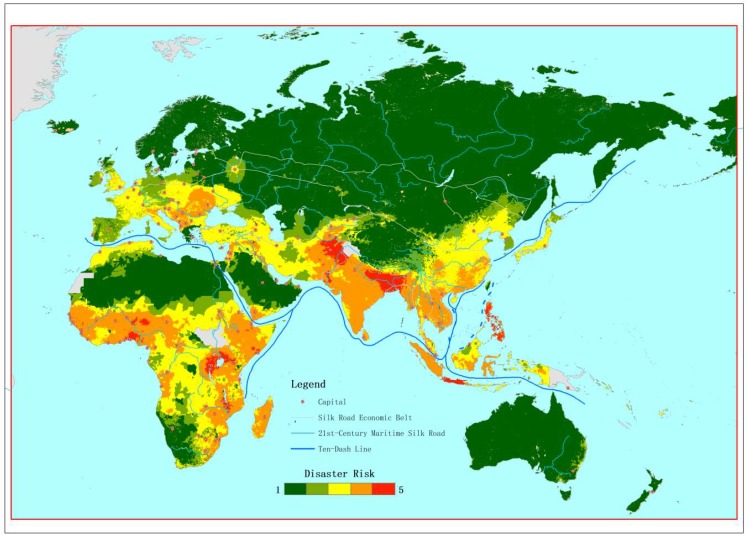
Health disaster risk of countries along the Belt and Road Initiative (BRI) region.

The final disaster risk index along the Belt and Road region were calculated by combining the proposed vulnerability index, the hazard index, and the exposure index. The final index was in pixel-based format, and therefore was presented in a world map for illustration (Figure 5). The top five areas with the highest disaster risk that was identified in this study were in locations near the Philippines, Afghanistan, Bangladesh, Somalia, and Indonesia. Meanwhile, northwest China, North Africa, eastern Europe, and Australia were found to have relatively lower risks.

## 4. Discussion

This paper presents a three-phase methodology approach for disaster risk assessment that incorporated health vulnerability dimensions into an existing hazard-based disaster risk map development. The proposed health vulnerability assessment index covers seven health dimensions, including infectious disease, chronic disease, maternity, under five years old, healthcare services, immunization, and the dependency ratio. Under these seven dimensions, nine indicators were used for formulating the vulnerability index, namely: (1) proportion of population below 15 years and above 65 years, (2) under-five mortality ratio, (3) maternal mortality ratio, (4) prevalence of tuberculosis, (5) the age-standardized raised blood pressure, (6) physician ratio, (7) hospital bed ratio, and (8) coverage of the MCV1 and (9) DTP3 vaccines. Then, the vulnerability index that was formed was combined with an existing disaster risks index from the Institute of Mountain Hazards and Environment at The Chinese Academy of Science.

Based on the formula established in this study and the public data mentioned in the Methods session, Greece, the Republic of Korea, and Belarus were found to be the three least vulnerable countries, while Somalia, the Central African Republic, and Chad were the three most vulnerable countries. After combining the vulnerability index with the exposure and hazard indexes, areas close to the Philippines, Afghanistan, Bangladesh, Somalia, and Indonesia were shown to have the highest disaster risk among the 147 study countries along the BRI region.

The Index For Risk Management (INFORM) and the World Risk Index are global disaster risk indexes that have incorporated health related components for vulnerability. INFORM is a global risk assessment index that is a collaboration of the Inter-Agency Standing Committee Task Team for Preparedness and Resilience and the European Commission and is adopted in the Global Risk Map (https://globalriskmap.terria.io/About.html) (including 190 countries). These included tuberculosis prevalence, HIV prevalence, malaria death rate, and under-five mortality as vulnerability indicators, and have included physician density as a capacity-coping indicator. The World Risk Index (171 countries considered) presented by Birkmann and Welle, and the Integrated Research on Disaster Risk (IRDR) team [19], have combined susceptibility, lack of coping, and adaptive capacity within vulnerability. They considered a dependency ratio for susceptibility, physicians, and hospital beds ratio for coping capacity, and private and public medical expenditure for adaptive capacity. Table 2 below compares the health components considered in the two mentioned indexes and those included in this index. The INFORM model and the Word Risk Index were built with sophisticated calculations and variables from different aspects in risk assessments such as health-related components, economic status, political environment, and infrastructure. Yet, many important health vulnerability burdens such as non-communicable diseases were not included.

The study aims to advance the current disaster risk assessment to include health vulnerability, which will inevitably affect population vulnerability in times of crisis. Thus, the discussion here focused on health-related components. The index proposed in this study has included indicator(s) of seven important health components in disaster risk assessment. Specifically, although chronic diseases have been cited as the most important causes of mortality and morbidity [31], attention has yet to be placed on global disaster risk assessment. People living with chronic diseases usually highly rely on long-term medicine for disease management. Unstable medicine access during and after disasters may lead to preventable adverse health consequences for the affected individual and the community. Considering the increasing prevalence of chronic diseases globally, related factors are suggested to be included as a vulnerability indicators in estimating disaster risks.

Table 3 shows the top 10 countries with the highest vulnerability/lowest coping capacity obtained from the three indexes. Despite the lack of consideration of the socio-economic, political, and infrastructural aspects and the different health components considered in the proposed index, five out of the 10 countries also appeared in top 10 from the other two indexes. This suggested that the health dimension is a strong determinant for disaster risk vulnerability. It is of note that although South Sudan was shown to be the most vulnerable with least coping capacity in the INFORM model, due to the missing data for South Sudan in the dataset that was used in this study (WHO and the World Bank), South Sudan was not included in this analysis. Ukraine was the most vulnerable country in Europe according to this study. Its vulnerability was mainly due to the country’s low vaccination rate, which was almost the lowest among all of the studied countries in the dataset. Since this study only accounted for health-related aspects calculating vulnerabilities, rather than other factors such as economic and political factors, Ukraine was found to be more vulnerable for disaster risk compared to the INFORM and the World Risk Indexes. The relatively higher coping capacity for European countries might reflect the better socio-economic status in these countries.

Vulnerability made substantial contributions to understandings and conceptualizations of disaster risk. When populations are exposed to natural disasters, vulnerable groups such as young children, older people, and people with mobility problems have more difficulties in evacuations, and might have a higher immediate risk of injuries. After extreme natural events, people might lose their homes and have to stay closely together in temporary shelters where hygiene and living conditions are usually compromised. In communities with low vaccination rates, outbreaks of infectious diseases might happen. Chronic diseases, as well as mental and psychological problems, also create health concerns and add extra stress to the healthcare system. Efficient medical services are important for handling immediate and indirect health needs in affected areas. Delayed or insufficient medical support to the affected people would increase fatality and morbidity. This could be due to the non-perfect healthcare system in local areas with poor coping capacity. Thus, this study proposed to include health vulnerability in estimating disaster risks. The results of this study have shown that populations with higher vulnerability were under higher overall risks than populations with lower vulnerability, given that they have comparable hazards and exposures. For example, both Japan and Bangladesh were prone to earthquakes (hazard), and were densely populated (exposure). However, after considering the vulnerability index proposed in this study, area near Bangladesh has a higher overall disaster risk than Japan.

The study has several limitations. Firstly, due to the study focus, vulnerability (including adaptive and coping capacity) related to other aspects such as sociodemographic and political aspects were not included for this specific model. Secondly, this study applied factor analysis in determining the underlying constructs of the selected predictors. It is important to highlight that factor analysis does not explain the cause of the convertibility [32]; the factors presented in this study were based on the understandings and experience in the field and the references considered. Despite the sample size that was used for this factor analysis being less than the common agreed size of 200 [32] due to the limited number of countries, it was larger than the suggested minimum size of 100 [32], which should provide considerable power for the analysis. Finally, the results presented were based on the 147 countries along the Belt and Road region; the reported vulnerability ranking is subject to change when different countries are considered.

Thirdly, this proposed index is highly driven by data availability and accuracy. Although the disability rate is another important health determinant in disasters that is advocated by WHO, due to the lack of data, disability was not included in this analysis. Similarly, this study can only include a few indicators as proxies for each health dimension due to the limitation of data. This study did not impute missing data due to simplicity and accuracy. Therefore, the number of missing data would be more than those used in the compared indexes. The accuracy of the results of this analysis was highly dependent on the accuracy of the open access data. Data from different organizations may not be consistent due to inconsistency in collection methods, study periods, calculations, imputation methods, or even data sources. Results should be read with caution.

In addition, although the use of all-hazard approach intended to include all disaster types for the hazard index, the health vulnerability index may face constrains in covering the whole disaster spectrum. For instance, physician density may be an important health indicator for coping capacity during disease outbreaks; however, it may be less relevant to injury risk and health vulnerability during and after tsunamis. 

According to the WHO, disaster risk management involving health components can avoid or reduce relevant health impacts [33]. While disaster risk assessment is one of the important components of risk management, hazard, vulnerability and capacity are the three elements that were most commonly considered in disaster risk assessment [33]. Among various dimensions of human vulnerability, low sociodemographic status, female gender, large dependency ratio, chronic diseases and disability are risk factors for disaster-associated mortality and morbidity [33]. Some current risk assessment indexes indicated vulnerability or coping capacity by using sociodemographic factors such as age, poverty, ethnic minority and education level [34,35] while some of them also included health-related variables [18,19]. 

However, not many existing global based disaster risk assessment model considered underlying non-communicable diseases patterns. A country specific vulnerability index for wildfire from the Environmental Protection Agency, the United State, considered chronic diseases such as asthma, diabetes, hypertension and obesity [36]. This study suggests the inclusion of chronic disease in addition to the health-variables considered in current disaster risk assessment tools (World Risk Index, INFORM) and demonstrated how health-related vulnerability could be added into existing risk assessment tools using a relative simple statistical method and open access data. The results of this study could be served as a basis for future development of disaster risk assessment model or adding a health related component to the existing one.

Specifically, the BRI counties are undergoing rapid socio-economic development. Lifestyle changes and westernized diets may increase the prevalence of chronic diseases such as diabetes and cardiovascular diseases [22]. The index presented in this paper may provide a more comprehensive health-related disaster risk assessment tool which may of the Belt and Road Imitative countries. This would help in improving Health-EDRM capacity planning, resources distribution and arrangement for the regions. 

## 5. Conclusions

This paper presents a health vulnerability index that aims to enhance disaster risk assessment for disaster risk reduction. The suggested health vulnerability index covers seven health vulnerability dimensions, including infectious disease, maternal mortality, under-five mortality, healthcare services, immunization, the dependency ratio, and chronic disease. This new index incorporated important health dimensions such as chronic diseases into the existing hazard-based disaster risk mapping approach.

Attention has to be paid specifically to the health vulnerability, which is associated with population living with chronic diseases. As more comprehensive health-related disaster risk assessments emerge, policy makers and program planners may engage in better resources and capacity planning, distribution, and arrangement to address the needs of Health-EDRM in the disaster-affected regions along the Belt and Road Initiative countries.

## Figures and Tables

**Table 1:** Key indicators of health vulnerability and their relevance.

Dimension of Health Vulnerability	Indicator	Conceptual Relevance to Health Vulnerability
Vulnerable age ^a^	1. Population ages 0–14 and population ages 65 and above (% of total)	Extreme age groups (children and elderly) are known to be more vulnerable to health risks and less likely to be resilient when a disaster strikes. This is an important component in the “dependency ratio”. They are more likely to accumulate post-disaster health and service needs.
Premature mortality ^b^	2. Under-five mortality rate (probability of dying by age five per 1000 live births)	Leading indicator of health in the United Nation (UN)’s Sustainable Development Goals (SDGs). It is closely linked to maternal health.
Preventable mortality ^b^	3. Maternal mortality ratio (per 100,000 live births)	Leading indicator of health in the UN’s Sustainable Development Goals (SDGs). In addition to preventable deaths, this indicator reflects the capacity of health systems to effectively prevent and address the complications occurring during pregnancy and childbirth.
Vaccination gap for measles ^b^	4. Measles-containing-vaccine first-dose (MCV1) immunization coverage gap among one-year-olds (%)	Standard Expanded Program on Immunization (EPI) for common preventable Childhood Communicable Diseases for children <one year old. Coverage may be used to monitor immunization services as well as guide disease eradication and elimination efforts, and are a good indicator of health system performance. MCV1: Measles is one of the most contagious and mortality-causing diseases in displaced camps. DTP3: Tetanus is common preventable infection associated with injury/wound.
Vaccination gap for diphtheria, tetanus, and pertussis ^b^	5. Diphtheria tetanus toxoid and pertussis (DTP3) immunization coverage gap among 1-year-olds (%)	
Chronic diseases status ^b^	6. Raised blood pressure (SBP ≥140 OR DBP ≥90), age-standardized (%)	A proxy indicator for chronic non-communicable disease. Hypertension and heart disease are some of the leading causes of mortality and morbidity globally. Disease status and potential activity limitations among adults can impair one’s ability to prepare, respond, or recover from a disaster.
Infectious disease ^b^	7. Incidence of tuberculosis (per 100,000 population per year)	Tuberculosis (TB) is the second leading infectious cause of death, and one of the most burden-inflicting diseases in the world. SDGs include ending the TB epidemic by 2030. The incidence of tuberculosis gives an indication of the burden of TB in a population.
Coping capacity ^b^	8. Hospital beds (per 10,000 population)	Health systems resources indicate the level of access to care and the provision of quality medical care, which are highly correlated with live-saving and health status.
	9. Physicians’ density (per 1000 population)	

Source: ^a^ Data collected from the World Bank; ^b^ Data collected from the World Health Organization. DBP: diastolic blood pressure; SBP: systolic blood pressure.

**Table 2:** Health-related components considered in the Index For Risk Management (INFORM) model, the World Risk Index, and the index developed in this study.

Components	INFORM	World Risk Index	The Proposed Index
Infectious diseases	Tuberculosis prevalence		Tuberculosis prevalence
	Estimate % of adults (>15) living with HIV		
	Malaria death rate		
Chronic diseases			Age-standardized raised blood pressure
Maternal outcome	Maternal mortality		Maternal mortality
Children under five	Under-five mortality		Under five mortality
	Malnutrition in children under five		
Medical services and access	Physician ratio	Physicians ratio	Physicians ratio
		Hospital beds ratio	Hospital beds ratio
	Per capita expenditure on private and public health care	Public medical expenditure; private medical expenditure	
Immunization	Measles immunization coverage		Coverage of two the MCV1 and DTP3 vaccine
Dependency ratio		Proportion of population <15 years old and >65 years old	Proportion of population <15 years old and >65 years old

**Table 3:** The top 10 countries with the highest vulnerability/lowest coping capacity from the INFORM model, the World Risk Index, and the proposed index developed in this study.

Top 10 Countries/Regions with Highest Vulnerability/Capacity	INFORM		World Risk Index	The Proposed Index
	Coping Capacity	Vulnerability	Vulnerability Including Susceptibility, Coping Capacities, and Adaptive Capacities	Vulnerability
1	**South Sudan**	**South Sudan**	**Chad**	**Somalia**
2	**Somalia**	**Somalia**	Eritrea	**Central African Republic**
3	**Chad**	**Central African Republic**	Afghanistan	**Chad**
4	**Central African Republic**	**Democratic Republic of the Congo**	Haiti	Equatorial Guinea
5	**Democratic Republic of the Congo**	**Chad**	**Niger**	Nigeria
6	Yemen	Yemen	**Central African Republic**	**Guinea**
7	Guinea-Bissau	Syria	Liberia	**Sierra Leone**
8	Eritrea	Afghanistan	**Sierra Leone**	Mali
9	Liberia	Haiti	Mozambique	**Niger**
10	Togo	Sudan	**Guinea**	**Democratic Republic of the Congo**
